# The Predictive Value of Individual Electric Field Modeling for Transcranial Alternating Current Stimulation Induced Brain Modulation

**DOI:** 10.3389/fncel.2022.818703

**Published:** 2022-02-22

**Authors:** Basil C. Preisig, Alexis Hervais-Adelman

**Affiliations:** ^1^Department of Psychology, Neurolinguistics, University of Zurich, Zurich, Switzerland; ^2^Donders Institute for Cognitive Neuroimaging, Radboud University, Nijmegen, Netherlands; ^3^Max Planck Institute for Psycholinguistics, Nijmegen, Netherlands; ^4^Neuroscience Center Zurich, Eidgenössische Technische Hochschule Zurich, University of Zurich, Zurich, Switzerland

**Keywords:** electric field modeling, transcranial alternating current stimulation (tACS), fMRI, connectivity, dichotic listening

## Abstract

There is considerable individual variability in the reported effectiveness of non-invasive brain stimulation. This variability has often been ascribed to differences in the neuroanatomy and resulting differences in the induced electric field inside the brain. In this study, we addressed the question whether individual differences in the induced electric field can predict the neurophysiological and behavioral consequences of gamma band tACS. In a within-subject experiment, bi-hemispheric gamma band tACS and sham stimulation was applied in alternating blocks to the participants’ superior temporal lobe, while task-evoked auditory brain activity was measured with concurrent functional magnetic resonance imaging (fMRI) and a dichotic listening task. Gamma tACS was applied with different interhemispheric phase lags. In a recent study, we could show that anti-phase tACS (180° interhemispheric phase lag), but not in-phase tACS (0° interhemispheric phase lag), selectively modulates interhemispheric brain connectivity. Using a T1 structural image of each participant’s brain, an individual simulation of the induced electric field was computed. From these simulations, we derived two predictor variables: maximal strength (average of the 10,000 voxels with largest electric field values) and precision of the electric field (spatial correlation between the electric field and the task evoked brain activity during sham stimulation). We found considerable variability in the individual strength and precision of the electric fields. Importantly, the strength of the electric field over the right hemisphere predicted individual differences of tACS induced brain connectivity changes. Moreover, we found in both hemispheres a statistical trend for the effect of electric field strength on tACS induced BOLD signal changes. In contrast, the precision of the electric field did not predict any neurophysiological measure. Further, neither strength, nor precision predicted interhemispheric integration. In conclusion, we found evidence for the dose-response relationship between individual differences in electric fields and tACS induced activity and connectivity changes in concurrent fMRI. However, the fact that this relationship was stronger in the right hemisphere suggests that the relationship between the electric field parameters, neurophysiology, and behavior may be more complex for bi-hemispheric tACS.

## Introduction

Transcranial electric stimulation (tES) is a type of non-invasive brain stimulation where relatively weak electric currents in the range of 1–2 mA are applied to a participant’s scalp. The most common forms are direct (tDCS) and alternating (tACS) current stimulation (Miniussi et al., 2013; Antal and Herrmann, 2016). The mechanism of action behind tDCS is alteration of the resting membrane potential in the subjacent cortex depending on the polarity of the applied current (Nitsche and Paulus, 2000; Ahn and Fröhlich, 2021). In contrast, tACS is thought to entrain the firing rate of neurons to the frequency of the alternating current (Herrmann et al., 2013; Reato et al., 2013). Dual-site tACS has been recently introduced as technique to manipulate the phase synchronization of local oscillations in two connected cortical areas with the aim to modulate the coupling of remote neural populations (Polanía et al., 2012, 2015; Helfrich et al., 2014; Alekseichuk et al., 2017, 2019; Saturnino et al., 2017; Meier et al., 2019; Misselhorn et al., 2019; Preisig et al., 2019, 2020, 2021; Reinhart and Nguyen, 2019; Schwab et al., 2019). Both tDCS and tACS have become very popular over the last two decades as they promise the possibility of causal inference about the functional role of stimulated brain regions and networks. Further, they are considered very safe, portable and relatively cheap brain stimulation methods (Antal et al., 2017).

Recently, tES has been criticized due to an apparent lack of replicability of the reported effects (Horvath et al., 2015a,b; Héroux et al., 2017; Veniero et al., 2017; Fekete et al., 2018; Guerra et al., 2020). Some authors have even questioned whether the applied field strengths are sufficient to induce neurophysiological effects (Lafon et al., 2017; Vöröslakos et al., 2018). Others found that behavioral effects attributed to tES interventions may be caused by indirect stimulation of the afferent nerves in the skin (Asamoah et al., 2019) or retina (Kar and Krekelberg, 2012). Although, some studies reported comparable effects of tDCS on motor and visual evoked potentials in mice (Cambiaghi et al., 2010, 2011) and humans (Nitsche and Paulus, 2000; Antal et al., 2004; Ahn and Fröhlich, 2021), effects identified in one species (e.g., rodents) or system (e.g., motor) can not always be translate as easily to another species (e.g., non-human primates or humans) or system (e.g., sensory systems or executive functions) (Beliaeva et al., 2021). One common limitation of tES studies in humans is that the assumed neurophysiological effects are not measured. Therefore, it is often difficult to differentiate whether the absence of a behavioral effect is the result of an absence of neuromodulatory effects in the targeted brain region or because the underlying neural mechanism for the behavior is different than hypothesized (Preisig et al., 2019). Moreover, it is known that there are inter-individual differences in the susceptibility for tES (Héroux et al., 2017; Guerra et al., 2020). One possible reason for individual variability in susceptibility to tES is the influence of individual anatomy and the resulting differences of the induced electric field inside the brain (Neuling et al., 2012; Opitz et al., 2018). Recent advances in the development of computational electric field models open the possibility to study these differences using simulations (Thielscher et al., 2015; Huang et al., 2017; Saturnino et al., 2017; Wischnewski et al., 2021). Using simulated electric fields revealed that the current practice of applying tES with a fixed montage and intensity results in highly variable electric field strengths in the target brain region (Kasten et al., 2019; Evans et al., 2020). However, it is still unclear to what extent variability in the electric field accounts for the variability of tES effects on behavioral and neurophysiological outcome variables.

**FIGURE 1:**
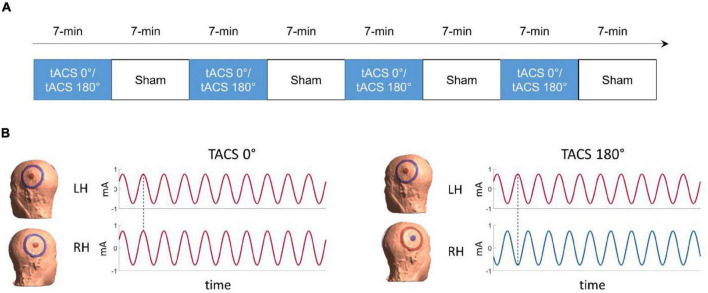
**(A)** Time-course of the experiment **(B)** illustration of the tACS conditions. On the left side, in-phase tACS with an interhemispheric phase lag of 0° (tACS 0°), on the right side anti-phase tACS with an interhemispheric phase lag of 180° (dotted line, tACS 180°). The colors represent the polarity (positive = red; negative = blue) of the current for the time stamp highlighted by the dotted line.

Phase synchronization of the activity of remote neural populations is hypothesized to be a key mechanism for functional connectivity among brain areas (Varela et al., 2001; Fries, 2005). In two recent studies (Preisig et al., 2020, 2021), we probed interhemispheric phase synchronization as a mechanism for acoustic feature binding using bi-hemispheric tACS. In both studies, we applied tACS with different interhemispheric phase lags: In-phase tACS (0° interhemispheric phase lag) and anti-phase tACS (180° interhemispheric phase lag) (Figure 1). The effects of tACS were quantified by comparison with a sham stimulation condition. A first study indicated that gamma tACS applied at 40 Hz changed the propensity of binaural integration of dichotic acoustic features (Preisig et al., 2020). In the second study (Preisig et al., 2021), we used concurrent functional magnetic resonance imaging (fMRI) to test whether the effect of gamma tACS results from changing the strength of functional connectivity between the left and the right auditory cortices. We found that gamma tACS reduced connectivity within the auditory cortices in both phase lag conditions, but only anti-phase tACS modulated interhemispheric brain connectivity. However, we only partially replicated the effect of gamma tACS on dichotic listening. Importantly, we found that inter-individual differences in the modulation of intra- and interhemispheric connectivity were correlated with auditory integration of dichotic stimuli: the stronger the induced connectivity reduction, the stronger the reduction in binaural integration.

In the present study, we investigated whether individual differences in the simulated electric field explain variability of online tACS effects. There are a few recent studies, which related the individual electric field to stimulation outcomes of tDCS (Antonenko et al., 2021; Mosayebi-Samani et al., 2021) and tACS (Kar et al., 2019; Kasten et al., 2019; Zanto et al., 2021). All studies reported a relationship between the strength of the individual electric field and neurophysiological (Kar et al., 2019; Kasten et al., 2019; Antonenko et al., 2021; Mosayebi-Samani et al., 2021) or behavioral (Zanto et al., 2021) outcome measures. In addition, Kasten et al. (2019) showed that also the precision of simulated electric field predicted tACS after-effects in magnetoencephalography (MEG) recordings. In the present study, we followed their analysis procedure to test whether the modeled precision and the maximum strength of the electric field can predict tACS induced changes in concurrent fMRI and auditory integration. Precision was determined as the spatial correlation between the electric field and the task evoked BOLD activity during sham stimulation. The maximum field strength was operationalized as the average field strength in the 10,000 voxels with largest electric field values in either gray and white matter compartments in each hemisphere. Here, we tested whether individual differences in the precision and the strength of electric field would predict the inter-individual variability in the tACS induced changes in the BOLD signal, interhemispheric connectivity, and dichotic listening.

## Materials and Methods

### Participants

Twenty-seven right-handed listeners with no history of hearing impairment (*M* = 21.89 years, *SD* = 3.14, 8 male) took part in a combined tES-MRI study. The present analysis is based on data that have been documented in a previous publication (Preisig et al., 2021). All participants had normal or corrected-to-normal visual acuity. The participants reported no history of neurological, psychiatric, nor hearing disorders. All participants had normal hearing (hearing thresholds of less than 25 dB HL at 250, 500, 750, 1,000, 1,500, 3,000, and 4,000 Hz, tested on each ear using pure tone audiometry) and no threshold difference between the left and the right ear larger than 5 dB for any of the tested frequencies. All participants gave written informed consent prior to the experiment. This study was approved by the local research ethics committee (CMO region Arnhem-Nijmegen) and was conducted in accordance with the principles of the latest Declaration of Helsinki.

### Electric Stimulation

Electric current was administered using two battery-driven transcranial current stimulators (Neuroconn, Ilmenau, Germany) using a custom set-up. The stimulators were placed in a shielded box including radiofrequency filters inside the faraday cage of the MR scanner. A two-way converter (A/D and D/A, Lindy) was used to convey the input signals for electric stimulation *via* optic cables from the scanner control room to the current stimulators in the shielded box.

Electric currents were applied through two high-density electrode configurations each consisting of concentric rubber electrodes: a central circular electrode (radius = 1.25 cm) and a surrounding ring electrode (inner radius = 3.9 cm, outer radius = 5.0 cm). Electrodes were kept in place with adhesive, conductive ten20 paste (Weaver and Company, Aurora, CO, United States). Each pair of center-surround electrodes was connected to a separate stimulator. The electrode configurations were centered according to the international 10–20 system over CP5 (above the left cerebral hemisphere) and CP6 (above the right cerebral hemisphere) (see Figure 1B). These scalp locations were chosen to produce relatively strong currents in the target regions over the auditory speech areas (i.e., left and right lateral superior temporal lobe) (Preisig et al., 2021).

Transcranial alternating current stimulation was applied at a frequency in the low gamma frequency band (40 Hz). Before starting the experiment, we ensured that the stimulation was well tolerated by all participants. Stimulation intensity was adjusted individually to the point at which the participant reported feeling comfortable or uncertain about the presence of the current (1.48 ± 0.06 mA, mean ± SD, peak-to-peak across participants). Impedance was kept below 10 kOhm. Stimulation was ramped up over the first and down over the final 6 s of each experimental block using raised-cosine ramps. In the sham condition, the onset ramp was followed immediately by an offset ramp of 6 s, that is, no electric stimulation was applied during the actual experiment. The ramp up and down was repeated at the end of the block.

### Experimental Design and Dichotic Listening Task

The experiment comprised eight task-based fMRI runs (four tACS and four sham runs) presented in pseudorandom order. tACS and sham runs were presented in alternating order with tACS being followed by sham (see Figure 1A) to reduce the risk of potential tACS after effects (Vossen et al., 2015). The order of tACS (*A* = tACS 0°, *B* = tACS 180°, *S* = sham) was either A-S-B-S-B-S-A-S or B-S-A-S-A-S-B-S and counterbalanced across participants. tACS was presented in two interhemispheric phase-synchronization conditions: In-phase tACS (0° interhemispheric phase lag) and anti-phase tACS (180° interhemispheric phase lag) between the central electrodes placed over the left and the right auditory speech areas (i.e., bilateral superior temporal lobe) (Preisig et al., 2021).

Each fMRI run consisted of 128 trials (88 dichotic stimuli and 40 trials without stimulus presentation). Each task fMRI run included 60 binaural integration trials where one ear was presented with an ambiguous syllable (intermediate between /da/ and /ga/) and the other with an acoustic feature (third formant, F3). The contralateral F3 could be low (2.5 kHz, consistent with /ga/) or high (2.9 kHz, consistent with /da/). If dichotically presented information is binaurally integrated, the F3 biases the perceived syllable. Participants reported, *via* button press, on every trial whether they heard a /da/ or a /ga/ syllable. In each of eight fMRI runs (∼7 min each) participants heard 30 high and 30 low F3 dichotic stimuli as well as 24 unambiguous control stimuli (12 times /da/ and 12 times /ga/). Four additional unambiguous stimuli were presented during the tACS ramping period. In unambiguous control stimuli, a clear syllable was presented to one ear and the F3 consistent with this syllable was presented to the other ear. Hence, unambiguous control stimuli could be readily interpreted based on monaural input.

For a detailed description of the task and the stimuli see Preisig and Sjerps (2019) and Preisig et al. (2020, 2021).

### Magnetic Resonance Imaging Acquisition and Pre-processing

Anatomical and functional MRI data were acquired on a 3-Tesla Siemens Prisma scanner using a 64-channel head coil. A 3-dimensional high-resolution T1-weighted anatomical volume was acquired using a 3D MPRAGE sequence with the following parameters: repetition time (TR) / inversion time (TI) / echo time (TE) = 2,300/1,100/3 ms, 8° flip angle, FOV 256 × 216 × 176 and a 1 mm × 1 mm × 1 mm isotropic resolution. Parallel imaging (iPAT = GRAPPA) was used to accelerate the acquisition resulting in an acquisition time of 5 min and 21 s.

Functional images were collected using a sparse acquisition protocol to minimize the impact of echo planar imaging (EPI) gradient noise during presentation of auditory stimuli (Hall et al., 1999). This was achieved by introducing a delay in TR of 2,070 ms during which the auditory stimuli were presented. For each participant and scanning run, 128 EPI volumes, each scan comprising 66 slices of 2 mm thickness were acquired using a interleaved acquisition sequence with multi-band acceleration (TR: 3,000 ms, TA: 930 ms, TE: 34 ms, flip angle: 90°, matrix size: 104 × 104 × 66, in plane resolution: 2 mm × 2 mm × 2 mm, Multiband accel. factor: 6×).

Pre-processing of MRI images was conducted in SPM12.^1^ including the following steps: (1) functional realignment and unwarping, (2) co-registration of the structural image to the mean EPI, (3) normalization of the structural image to a standard template, (4) application of the normalization parameters to all EPI volumes, and (5) spatial smoothing using a Gaussian kernel with a full-width at half maximum of 8 mm.

### Univariate Functional Magnetic Resonance Imaging Analyses

Voxel-wise BOLD activity was modeled in subjects’ native image space using realigned and unwrapped EPI images by means of a single subject first-level General Linear Model (GLM). For the univariate analysis, the design consisted of one regressor for auditory stimulus onset. For each run, six realignment parameters to account for movement-related effects and a constant term per functional imaging run were included in the model.

To identify brain regions that responded significantly to auditory stimuli and the task, T-contrasts (all auditory stimuli > implicit baseline) were computed for sham runs. To examine the tACS induced BOLD-response modulations, parameter estimates from tACS runs were contrasted with sham runs in each subject. Since, the design included 4 sham runs, but only two tACS runs per condition per participant, the two tACS runs were contrasted with all $\left(\begin{array}{c} n\\ k \end{array}\right)$ possible combinations of sham runs (where *n* = 4 and *k* = 2). The resulting six statistical *t*-maps per tACS condition were subsequently averaged into one *t*-map per tACS condition.

Transcranial alternating current stimulation induced BOLD signal modulation was computed as the average modulation strength in each hemisphere. For this purpose, we used brain masks from the laterality toolbox (Wilke and Lidzba, 2007) that comprised the entire left or right hemisphere, i.e., without excluding midline voxels. These masks were converted into the participant’s native space using the inverse normalization parameters. For subsequent analyses, *t*-values of mean difference in the BOLD signal between tACS (tACS 0°; tACS 180°) condition and sham stimulation was extracted from left and right hemisphere ROI.

### Functional Connectivity (Conn)

To investigate functional connectivity between the left and the right hemisphere in each tACS condition (tACS 0°; tACS 180°; sham) connectivity analysis was performed using the CONN toolbox (Whitfield-Gabrieli and Nieto-Castanon, 2012).^2^ The CONN toolbox is able to perform region of interest (ROI)-to-ROI functional correlation analysis according to the low-frequency, temporal fluctuations of BOLD signals.

In contrast to the univariate fMRI analyses, normalized and smoothed images were used for the functional connectivity analyses. We specified one regressor per tACS condition (tACS 0°; tACS 180°; sham) coding the onset of each stimulus. The BOLD signal of white matter and CSF, and the six realignment parameters to account for movement-related effects were used as covariates to remove unwanted physiologic and motion artifact effects. Additional pre-processing included de-noising with voxelwise removal of linear trends over each participant’s fMRI dataset and temporal filtering (bandpass 0.01 and 0.1 Hz).

First-level analyses included a weighted GLM for estimation of the bivariate correlation between the left and right hemisphere. As ROI, we used again the brain masks from the laterality toolbox (Wilke and Lidzba, 2007) that comprised the entire left or right hemisphere. For this analysis, ROI timeseries were extracted as the first eigenvariates from all voxels inside the ROI. For subsequent analyses, Fisher (arc-sine hyperbolic) –transformed coefficients of the ROI-to-ROI functional correlations were extracted for each condition (tACS 0°; tACS 180°; sham) and tACS (tACS 0°; tACS 180°) induced connectivity modulations were calculated relative to sham stimulation (Δ tACS–sham).

### Electric Field Modeling

We used the simnibs toolbox (Thielscher et al., 2015) for individualized electric field modeling. In a first step, headreco (Nielsen et al., 2018) was applied to build individual head models from participants’ native space T1-weighted images. Afterward, we used simnibs MATLAB functions to compute individual electric field calculations: Individual simulations of the electric fields induced by two central circular electrodes (radius = 1.25 cm) placed over CP5 and CP6, each surrounded by a concentric ring electrode (inner radius = 3.9 cm, outer radius = 5.0 cm). Because the in-phase and the anti-phase tACS condition results in slightly different electric fields, we performed the simulations separately for each condition. In the in-phase condition, the electric field was simulated such that the current in CP5 and CP6 was + 0.75 mA (amplitude of the current half-wave), while the current in the surrounding ring electrodes was −0.75 mA. The anti-phase condition was simulated such that the current was + 0.75 mA in CP5 and the ring electrode surrounding CP6, while the current was −0.75 mA in CP6 and the ring electrode surrounding CP5 (see Figure 1B). Two participants preferred to lower stimulation to an intensity level of 1.3 mA peak-to-peak. For those participants we used a corresponding current value of ± 0.65 mA for the simulation.

### Data Analysis

We computed two measures of the inter-individual variability spatial PRECISION, i.e., how well does the electric field overlap with the targeted brain activity, and STRENGTH, i.e., the maximal electric field inside the brain (Kasten et al., 2019). To account for putative inter-hemispheric difference in precision and strength of the electric field, both indices were calculated separately for the right and the left hemisphere. As for the univariate fMRI and the connectivity analysis, we used the left and right hemisphere mask from the laterality toolbox (Wilke and Lidzba, 2007) to extract the relevant voxels from simulated electric fields.

**FIGURE 2:**
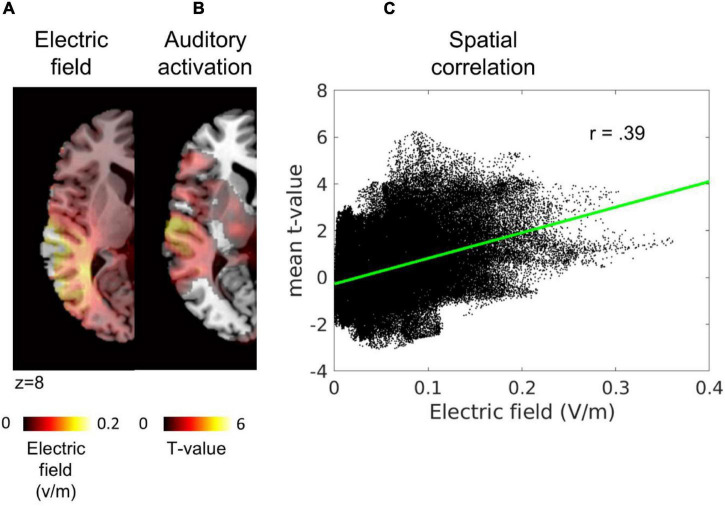
Illustration TACS precision estimation. From left to right, **(A)** simulation of the electric field in a representative participant, **(B)** task evoked BOLD activity during sham stimulation in the same participant, and **(C)** the spatial correlation between the two variables. Each black dot in the scatter represents one voxel: *x*-axis = the simulated electric field value, *y*-axis = mean *t*-value from the contrast auditory > baseline. In red, the least-square line of the correlation. Thresholded maps were used for visualization purposes in panels **(A,B)**. As can be seen in panel **(C)**, the whole map was used to compute the spatial correlation.

Following the procedure described by Kasten et al. (2019) precision was calculated as the spatial Pearson correlation between the electric field and the task-evoked BOLD activity during sham stimulation in each hemisphere (see Figure 2). To this end, data were projected onto an equally spaced 1-mm grid in the participant’s native image space using FieldTrip function ft_sourceinterpolate (Oostenveld et al., 2010).

As an index for the strength of the electric field inside the brain, we identified the 10,000 voxels with largest electric field values within gray and white matter compartment in each hemisphere and computed the average electric field magnitude across these voxels per hemisphere.

To investigate whether precision and strength of the electric field can account for individual differences in TACS induced BOLD signal modulation, brain connectivity changes, and behavioral changes in dichotic listening performance, we computed separate repeated measures Analysis of Covariance (ANCOVA) for these dependent variables including precision and strength as covariates. Statistical analyses were conducted in R (Version 4.1.1).

## Results

### Individual Electric Fields

**FIGURE 3:**
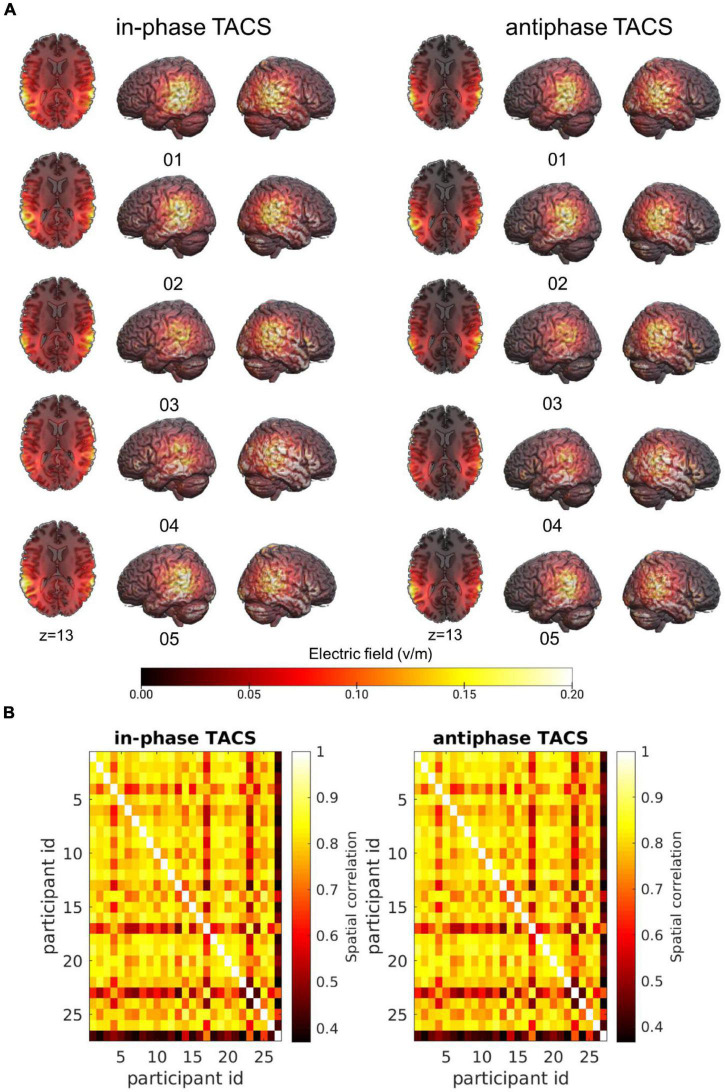
Individual variability of the electric field across participant. **(A)** Simulations of the electric fields with concentric ring electrodes centered over CP5 and CP6 (see section “Materials and Methods”) applied at 1.5 mA (peak-to-peak) illustrated for five participants in each tACS condition (tACS 0°; tACS 180°). Simulations were performed on the native image space of the participant and warped into MNI space for visualization purposes. **(B)** Spatial Pearson correlations between the electric fields of individual participants separated by tACS condition.

As expected, we found differences across subjects in terms of peak electric field magnitude inside the cortex as well as in the spatial distribution of electric fields (Figure 3A).

To characterize the similarity of electric fields across participants, individual simulation results were warped into Montreal Neurological Institute (MNI)-space. Spatial correlations of the fields were computed between all subjects separately for each Stimulation condition (tACS 0°; tACS 180°) to attain insights into the overall variability of the electric fields across participants. On average, electric fields correlated with *M_*tACS* 0°_* = 0.75 ± *SD_*tACS* 0°_* = 0.12 (*r*_*min*_ = 0.37, *r*_*max*_ = 0.89) and *M_*tACS* 180°_* = 0.75 ± *SD_*tACS* 180°_* = 0.12 (*r*_*min*_ = 0.37, *r*_*max*_ = 0.89) (Figure 3B).

We then examined the similarity between the simulated electric fields in the tACS 0° and tACS 180° within participants. The average spatial correlation across stimulation conditions (tACS 0°; tACS 180°) was high *M_*tACS* 0°_, _*tACS* 180°_* = 0.996 ± *SD_*tACS 0*^°_, _*tACS* 180°_* = 0.0025 (*r*_*min*_ = 0.99, *r*_*max*_ = 1). Given the large correlation between tACS 0° and tACS 180°, we averaged the electric fields across tACS conditions and computed one estimate of tACS precision and strength for the subsequent analyses.

The average electric field strength (average over strongest 10,000 voxels) was 0.13 V/m ± 0.03, mean ± SEM (min = 0.08 V/m, max = 0.19 V/m). This is comparable to the field strength reported by Kasten et al. (2019) who reported an average field strength of 0.13 V/m (min = 0.08 V/m, max = 0.36 V/m).

The average correlation was *M_*EF*,_ _*fMRI*_* = 0.12 ± 0.12 (*r*_*min*_ = −0.19, *r*_*max*_ = 0.31). The spatial correlations between task evoked BOLD activity and the simulated electric field were lower than the average correlation reported by Kasten et al. (2019) *M* = 0.55 for MEG data. Even if the reported average correlation was smaller in our study, correlations between the BOLD activity maps and the electric field maps were significantly above chance in all participants at *p* < 0.01 in both tACS conditions.

### The Impact of Individual Electric Field Strength on Induced BOLD Signal Changes

**FIGURE 4:**
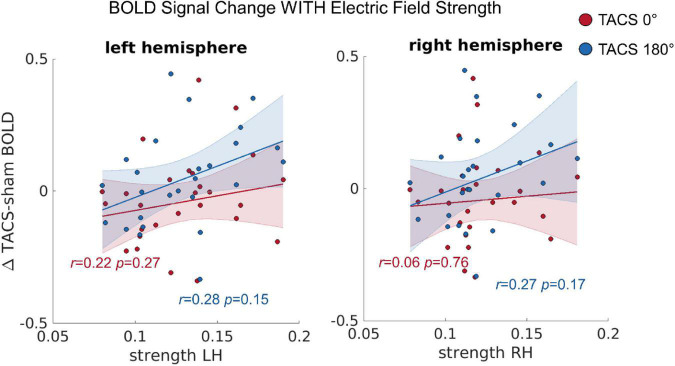
The correlation between the tACS induced BOLD signal change and the individual electric field strength in LH and RH. *r* = Pearson correlation. Error ribbon represents the 95% confidence interval obtained for the fit of a robust linear regression.

To test whether the observed inter-individual differences in the simulated electric fields predict the tACS-induced differences in BOLD signal (see section “Univariate Functional Magnetic Resonance Imaging Analyses”), we computed a repeated measures Multivariate Analysis of Covariance (MANCOVA) including the dependent variables BOLD change left hemisphere (LH) and BOLD change right hemisphere (RH), the covariates Precision LH, Precision RH, Strength LH, and Strength RH, as well as the within subject factor Stimulation Condition (tACS 0°; tACS 180°). Using Pillai’s trace we found statistical trends for significant effects of Strength LH on BOLD change LH [*V* = 0.21, *F*_(2,21)_ = 2.726, *p* = 0.089] and Strength RH on BOLD change RH [*V* = 0.24, *F*_(2,21)_ = 3.387, *p* = 0.053] (Figure 4), but no evidence for an effect of precision in either hemisphere. In contrast to a previous report, where we found that the BOLD modulation (Δ tACS–sham) induced by tACS 180° was significantly larger than the modulation induced by tACS 0° in the right posterior temporal sulcus (pSTS), we saw no evidence for similar effect of tACS phase here. The principal difference between the previously presented analysis (Preisig et al., 2021) and that presented here is the size of the ROI (30–33 vs. 80,352–80,787 voxels). Averaging over such a large region reduces sensitivity to effects occurring in only a circumscribed subregion of the ROI.

### Individual Electric Field Strength Predicts Connectivity Changes

**FIGURE 5:**
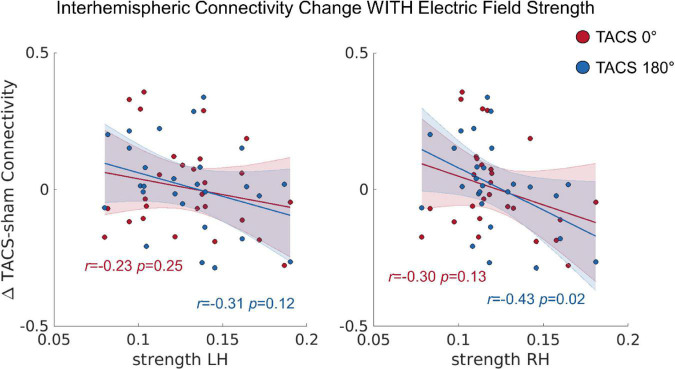
The correlation between the tACS induced connectivity changes and the individual electric field strength in LH and RH. *r* = Pearson correlation. Error ribbon represents the 95% confidence interval obtained for the fit of a robust linear regression.

In a next step, we tested whether the observed inter-individual differences in the simulated electric fields would also predict the tACS-induced connectivity changes (see section “Functional Connectivity (Conn)”), Here, we computed a repeated measures ANCOVA including the dependent variables Interhemispheric Connectivity Change, the covariates Precision LH, Precision RH, Strength LH, and Strength RH as well as the within subject factor Stimulation Condition (tACS 0°; tACS 180°). The covariate Strength RH was significantly related to the Interhemispheric Connectivity Change, *F*_(1,22)_ = 4.915, *p* = 0.037, *η_*p*_*^2^ = 0.18 (Figure 5). In contrast to our previous study, where we found that tACS 180° significantly reduced bi-directional interhemispheric effective connectivity between the auditory cortices, we found no effect of tACS phase in the current analysis. Note that this discrepancy might be related to the larger ROI chosen for the current analysis and the different connectivity measure.

### No Direct Influence of the Electric Field on Dichotic Listening

Finally, we tested whether the observed inter-individual differences in the simulated electric fields would predict the tACS induced changes in the dichotic listening performance (Stimulus discrimination, *d*-prime). Again, we computed a repeated measures ANCOVA including the dependent variables Interhemispheric Connectivity Change, the within subject factor Stimulation Condition (tACS 0°; tACS 180°) as well as the covariates Precision LH, Precision RH, Strength LH, and Strength RH. This time none of the covariates in the ANCOVA model predicted a significant portion of the variance in dichotic listening performance. We reasoned that there might be an indirect effect of electric field variability on dichotic listening performance by the induced neurophysiological changes. Indeed, we found a significant correlation between Interhemispheric Connectivity Change and Dichotic Listening in the anti-phase tACS condition (*Pearson r* = 0.42, *p* = 0.031). This finding is consistent with the relation that we reported in our previous study (Preisig et al., 2021): the stronger the induced connectivity reduction, the stronger the reduction in binaural integration. However, we found no comparable correlation of Dichotic Listening with Interhemispheric Connectivity Change in the in-phase condition (*Pearson r* = 0.24, *p* = 0.220), nor with BOLD signal changes in the LH (*r*_tACS 0°_ = 0.16, *p* = 0.440; *r*_tACS 180°_ = 0.22, *p* = 0.272) and RH (*r*_tACS 0°_ = 0.22, *p* = 0.270; *r*_tACS 180°_ = 0.22, *p* = 0.272).

## Discussion

### Summary Key Findings

In the current study, we aimed to establish a dose-response relationship between individual differences in electric fields and tACS induced brain activity and connectivity changes. We used computational modeling to simulate the electric fields of individual participants based on their anatomical brain scans. From these simulations, we derived two predictor variables: maximal strength (average of the 10,000 voxels with largest electric field values) and precision of the electric field (spatial correlation between the electric field and the task evoked brain activity during sham stimulation). Our results show considerable variability in the individual strength and precision of the electric fields. Importantly, the strength of the electric field over the right hemisphere predicted individual differences of tACS induced connectivity changes. Moreover, we found in both hemispheres a statistical trend for the effect of electric field strength on tACS induced BOLD signal changes. These findings are consistent with previous studies, which found a relationship between the tES field strength and neurophysiological outcome measures (Kar et al., 2019; Kasten et al., 2019; Antonenko et al., 2021; Mosayebi-Samani et al., 2021). In contrast, the precision of the electric field did not predict any neurophysiological measure. Further, neither strength, nor precision predict interhemispheric integration. In the following sections, we will discuss and interpret these results in the context of the current literature.

### Changes in Dichotic Listening Performance Are Correlated With Induced Connectivity Changes

We found no direct relationship between the strength or precision of the electric field and the behavioral response of the participant in dichotic listening (Stimulus discrimination, *d*-prime). However, we identified an indirect effect of the electric field strength over the right hemisphere *via* the induced interhemispheric connectivity changes on dichotic listening. This effect was restricted to the anti-phase tACS condition. This is consistent with our previous study (Preisig et al., 2021) where we found that only anti-phase tACS selectively modulated interhemispheric brain connectivity between the auditory cortices. Further, this supports a mechanistic model where tACS over auditory regions induces neurophysiological changes like BOLD signal modulations (Zoefel, 2018; Preisig et al., 2021) and electrophysiological changes (Rufener et al., 2019; Marchesotti et al., 2020), which are related to auditory perceptual changes.

### Precision Did Not Predict Neurophysiological Changes

Here, we showed that individual electric field strength in the right hemisphere explains part of the variability of the tACS effect on brain activity and connectivity changes. One may ask why the observed effect was stronger in the right hemisphere. The lateralization of the effect might be related to the design of the study. In our dichotic listening task, the disambiguating acoustic feature (third formant, F3) was presented to the left ear. Although the auditory nerve projects from each ear to both cerebral hemispheres, processing of acoustic input is initially dominant in the neural pathway, including the auditory cortex that is contralateral to the ear of presentation (Kimura, 1967; Sparks and Geschwind, 1968; Pollmann et al., 2002); for reviews see Westerhausen and Hugdahl (2008) and Hugdahl and Westerhausen (2016). In our case, initial right hemisphere processing follows left ear presentation. This means that in the context of the current task initial auditory processing in the right hemisphere could be more relevant to the task. Consistent with this, the effect of tACS on the BOLD signal in our previous study (Preisig et al., 2021) tended to be larger in the right hemisphere. This finding extends previous results by Kasten et al. (2019) suggesting that in the context of a task, the magnitude of the tACS effect is likely to be modulated by the task relevance of the targeted brain region. Individual variability (spatial correlation of the electric field across participants) and strength of the electric fields were comparable to the results reported by Kasten et al. (2019). However, the average precision of the electric fields was significantly lower in the present study [*r* = 0.12, compared to 0.55 in Kasten et al.’s (2019) report] and did not predict changes in our neurophysiological outcome variables. There are numerous potential explanations for this, which are likely related to difference between the two studies. One difference between the two studies is that they targeted different perceptual modalities. The visual cortex stimulated by Kasten et al. (2019) is presumably easier to target with tES than the auditory cortical regions targeted in the present study. The visual cortex is larger than the auditory cortex. Moreover, the primary visual cortex is located on the cortical surface, while the primary auditory cortex is hidden in the sylvian fissure. Another difference is the neuroimaging modality. In contrast to studies, which recorded electrophysiological data (Kasten et al., 2019; Zanto et al., 2021), we cannot confidently assert based on fMRI data that oscillatory gamma power and gamma band synchronization of respective cortical areas were affected. Second, our neurophysiological measures (BOLD signal and functional connectivity modulations) do not allow us to state that the effect was frequency specific and confined to the gamma frequency band. Nevertheless, there is evidence that gamma band modulations co-localize with BOLD signal in humans (Lachaux et al., 2007). Moreover, a recent study found a link between vasodilation, interhemispheric connectivity and neural gamma band activity in mice (Mateo et al., 2017). Another difference between the two studies is that they applied different tACS frequencies. Kasten et al. (2019) applied alpha tACS, while we applied gamma tACS in the current study. There is evidence that higher tACS frequencies like gamma are less effective than lower frequencies like alpha. This is because of the low-pass properties of neuronal membranes which attenuate high frequency stimulation (Deans et al., 2007; Esmaeilpour et al., 2020; Thiele et al., 2021). Further, it should be acknowledged that offline tACS effects like the ones reported by Vossen et al. (2015) and Kasten et al. (2019) may be caused by different neurophysiological mechanisms like spike-timing-dependent-plasticity (Herrmann et al., 2013), whereas online tACS effects like the ones reported in the present study are more likely caused by neural entrainment (Reato et al., 2013; Krause et al., 2019; Johnson et al., 2020). Finally, the two studies applied a different electrode montage, while Kasten et al. (2019) applied a classical bipolar montage, we applied a dual-site HD montage. In general, the higher focality of HD tES has the advantage that less brain tissue of no-interest is stimulated (Edwards et al., 2013; Kuo et al., 2013). However, previous research indicates that the higher focality of HD tES comes at the cost of higher interindividual electric field variability (Mikkonen et al., 2020). In our study, individual variability of the electric field, quantified as the spatial correlation across individual participants, was comparable to Kasten et al. (2019), who used a classical bi-polar montage. However, the low average precision and the high variability of this measure in our study indicates that HD-tACS centered on fixed electrode positions might by suboptimal in terms of tES precision. Our results thus provide evidence for the potential value of employing individualized HD montages to enhance effectivity of focal tES stimulation in future studies.

### What Is the Required Field Strength

What is the required tES field strength to modulate neuronal activity in humans? To address this question electric fields simulated in the human brain are often compared against thresholds identified in animal research. These thresholds are usually in the range from 0.2 to 0.5 V/m (Fröhlich and McCormick, 2010; Reato et al., 2013). It should be noted that there are some differences between the experimental designs employed in animals and humans, which may limit the translation of these voltage thresholds from animals to humans. In animal research, tES is often applied to *in vitro* brain slices, or if applied *in vivo*, intracranial stimulation is applied to local neuron assemblies. Moreover, stimulation is usually administered over short time periods in the range of a few seconds. In contrast, tES in human is often applied to relatively large brain regions *via* scalp electrodes and over a time span of several minutes. This means that stimulation effects in humans may build up over longer periods of time spanning larger brain networks. In line with previous research (Kasten et al., 2019), our simulations suggest an electric field strength of 0.1–0.2 V/m. Even though this field strength is lower than the field strength reported in animals, we find a relationship between the magnitude of the electric field and changes in functional connectivity between hemispheres. This indicates that these field strengths might be sufficient to modulate functional connectivity under the given combination of stimulation parameters and the experimental task. However, further research in this field is necessary to draw conclusions because the observed neural modulations are small.

### Limitations

This study employed computational modeling to obtain an estimate of the individual electric field inside the brain. This approach brings a degree of uncertainty and constitutes a simplification of the ground truth. For example, the accuracy of the estimated electric field can be influenced by errors in automatic tissue segmentation. Recent attempts to validate the results of electric field modeling show that the spatial distribution of the electric field is usually well predicted, while there is a trend to overestimate the electric field strength (Opitz et al., 2016, 2018; Huang et al., 2017; Krause et al., 2019). For our purposes, the exact field strength was less important than the relative difference between individual participants. Therefore, we consider these simulations adequate for the purpose of the current investigation. However, other sources of variability like thickness of the electrode paste or the positioning of the electrodes can affect the strength and precision of the electric field as well. Unfortunately, these factors vary from participant to participant and are therefore difficult to take into account in the modeling process (Saturnino et al., 2015). With regard to the effectiveness of tACS, the predictive value of the electric field parameters like strength and the precision are limited because they do not account for non-linear relationship between tACS frequency and tACS effectiveness (Deans et al., 2007; Esmaeilpour et al., 2020; Thiele et al., 2021). Thus, further validation and optimization of the applied computational models is required to increase confidence and accuracy of their predictions.

## Conclusion

In the current study, we translated an analysis pipeline which has been recently describe for tACS after effects in MEG data to concurrent tACS-fMRI. We find evidence for the dose-response relationship between individual differences in electric fields and tACS induced connectivity changes in concurrent fMRI. However, the fact that this relationship was limited to the right hemisphere suggests that the relationship between the electric field parameters, neurophysiology, and behavior may be more complex for HD dual-site TACS.

## Data Availability Statement

Publicly available datasets were analyzed in this study. This data can be found here: https://doi.org/10.34973/dt33-sj34.

## Ethics Statement

The studies involving human participants were reviewed and approved by the Local Research Ethics Committee (CMO region Arnhem-Nijmegen). The patients/participants provided their written informed consent to participate in this study.

## Author Contributions

BP performed the statistical analysis and wrote the first draft of the manuscript. Both authors contributed to conception and design of the study and contributed to manuscript revision, read, and approved the submitted version.

## Conflict of Interest

The authors declare that the research was conducted in the absence of any commercial or financial relationships that could be construed as a potential conflict of interest.

## Publisher’s Note

All claims expressed in this article are solely those of the authors and do not necessarily represent those of their affiliated organizations, or those of the publisher, the editors and the reviewers. Any product that may be evaluated in this article, or claim that may be made by its manufacturer, is not guaranteed or endorsed by the publisher.

## Footnotes

1
http://www.fil.ion.ucl.ac.uk/spm

2
www.nitrc.org/projects/conn
